# Supplementary material for: An Insight into the Sorption Behavior of 2,3,7,8-Tetrachlorodibenzothiophene on the Sediments and Paddy Soil from Chaohu Lake Basin
Source: Int J Environ Res Public Health. 2022 Sep 9;19(18):11346. doi: 10.3390/ijerph191811346 (PMC9517350; doi:10.3390/ijerph191811346)
Supplement: Supplementary file 1 [file ijerph-19-11346-s001.zip › ijerph-1847468-supplementary.pdf]

Supplementary Materials

An Insight into the Sorption Behavior of 2,3,7,8-Tetrachlorodibenzothiophene on the Sediments and Paddy Soil from Chaohu Lake Basin

Kainan Nian [1,2,†], Wenli Xiong [1,2,†], Yalu Tao [1], Ziqing Zhu [3], Xiaoxue Pan [1,2,*], Kang Zhang [1] and Xuesheng Zhang [1,2,*]

[1] School of Resources and Environmental Engineering, Anhui University, Hefei 230601, China; nkn19971226@163.com (K.N.); xiongwenli1018@163.com (W.X.); 13855596077@163.com (Y.T.); zhangkang20020911@163.com (K.Z.)

[2] Laboratory of Wetland Protection and Ecological Restoration, Anhui University, Hefei 230601, China

[3] China Science and Technology Intelligent Agricultural Valley Collaborative Innovation Research Institute, Hefei 231131, China; zzq2023jd@163.com

[*] **Author for correspondence:**

panxiaoxue1208@163.com (X. P.); zhangqing8725@163.com (X. Z.)

† These authors contributed equally to this work.

Figures: 2

Tables: 2

Total pages: 12

Title **Page**

Determination of the basic sediments and soil properties S-3

Standard solution configuration and standard curve plotting of 2,3,7,8-tetra-CDT S-5

Freundlich adsorption isotherm S-6

Figures legends

Figure S1. The sampling sites in Chaohu Lake (S1-S3) and the paddy soil (T1). S-7

Figure S2. The correlations between f_{oc} and logK_{OC} of different sediments and soil. S-8

Table contents

Table S1. Occurrence of PCDTs in various environmental matrices. S-9

Table S2. The method detection limits (MDLs) and recoveries of 2,3,7,8-TCDT in sediments and soil. S-11

References S-12

Determination of the basic sediments and soil properties

(1) Soil organic carbon.

Potassium dichromate oxidation spectrophotometry was used for the determination of soil organic carbon. A pipette was applied to transfer 25.00 mL of 0.4 mol/L (1/6$K_2Cr_2O_7$) reference solution into a 250 mL triangular flask. Subsequently, 3-5 drops of indicator of endorphin were added and titrated with 0.5 mol/L $FeSO_4$ solution to the endpoint (gray-green to brick red). Each group of experiments was conducted in triplicates, and the solution was used after the accurate concentration of $FeSO_4$ was calculated.

About 0.5 g of the air-dried soil sample was accurately weighed and placed into a 500 mL triangular flask. Afterward, 10 mL of 1 mol/L (1/6 $K_2Cr_2O_7$) solution was accurately added to the soil sample, and the bottle was rotated to mix evenly. Then, 20 mL of concentrated sulfuric acid was added, and the blank control was determined without the soil sample. The triangle flask was slowly rotated for 1 min and placed on an asbestos plate for about 30 min. After cooling down to room temperature, the solution was diluted to 250 mL with water, in which 2 ~ 3 drops of endorphin indicator were added, and titrated with 0.5 mol/L $FeSO_4$ standard solution to the endpoint. At the endpoint, the solution color was changed from green to dark green, and $FeSO_4$ is gradually added until brick red is formed. Three parallel experiments were conducted.

(2) Soil texture.

A laser particle size analyzer was used to determine the soil texture. About 15 g of soil samples were weighed, and the exact mass was recorded. 45 mL 3% HMP (hmethylotriamine) was added to disperse the soil particles (ratio: 1:3), which were then shaken on a round trip shaker for 2 h (120 times/min). After dispersion, the mud was screened with a 0.053 mm screen to separate the sand particles. The sand was dried at 55 ℃ until its mass is constant. The solution containing powder and clay particles was transferred to a 1000 mL beaker and

stirred to form a suspension, followed by a stand at room temperature (18-24 ℃) for 1.5-6 h. After settling, the suspended clay particles were partially dumped, and the remaining powder particles were dried in a beaker at 105 ℃ until the mass is constant, and then weighed.

(3) Total nitrogen.

The total nitrogen of soil was determined using the Kjeldahl method. 0.500 g soil sample is accurately weighed and added to the Kjeldahl bottle containing 1.5 g reductive mixed catalyst. And then, add 4 ml concentrated H_2SO_4, heat and boil in fume hood for about 1.5 h, and cool until the contents are clear. Distillation was carried out in a still with 2% boric acid solution as the receiving solution. After distillation, the solution was titrated to pink with 0.01 mol/L HCl standard solution. Three balance tests and a blank control group should be performed.

(4) pH.

The pH of the soil suspension was measured using the potentiometry method. Briefly, 10 g of soil was put into a 100 mL beaker and then 50 mL distilled water (bubbling nitrogen for 1 h to remove carbon dioxide) was added. The suspension was shaken for 30 min at 180 rpm and left for 30 min. Then, a pH meter (S20, Mettler-Toledo) was used to determine the pH of soil suspension.

Standard solution configuration and standard curve plotting of 2,3,7,8-tetra-CDT

2,3,7,8-tetra-CDT was accurately weighed at 50 mg, and a 25 mL volumetric bottle was used to prepare a 2 mg/mL sample reserve solution, which was fixed in methanol for use. Six 25 mL volumetric bottles were prepared, and the sample reserve solution of a certain volume was accurately removed by pipette, and diluted with methanol into 6 standard solutions with the concentration gradient. The configured series of standard solutions were transferred to a brown measuring bottle and determined by the HPLC. According to the standard solution concentration and the corresponding peak area, the corresponding standard curve was drawn.

Freundlich adsorption isotherm

Freundlich adsorption isotherm is as follows:

$$q=k_f c^{1/n} \quad \text{(S1)}$$

k_f, adsorption constant

n, constant, it is generally believed that when $1/n$ is between 0.5 ~ 1.0, the substance is easy to adsorb; in the case of $1/n>2$, the substance is hard to adsorb. Taking the logarithm of both sides of Equation (1), we can get:

$$lgq = \frac{1}{n} lgc + lg\, k_f \quad \text{(S2)}$$

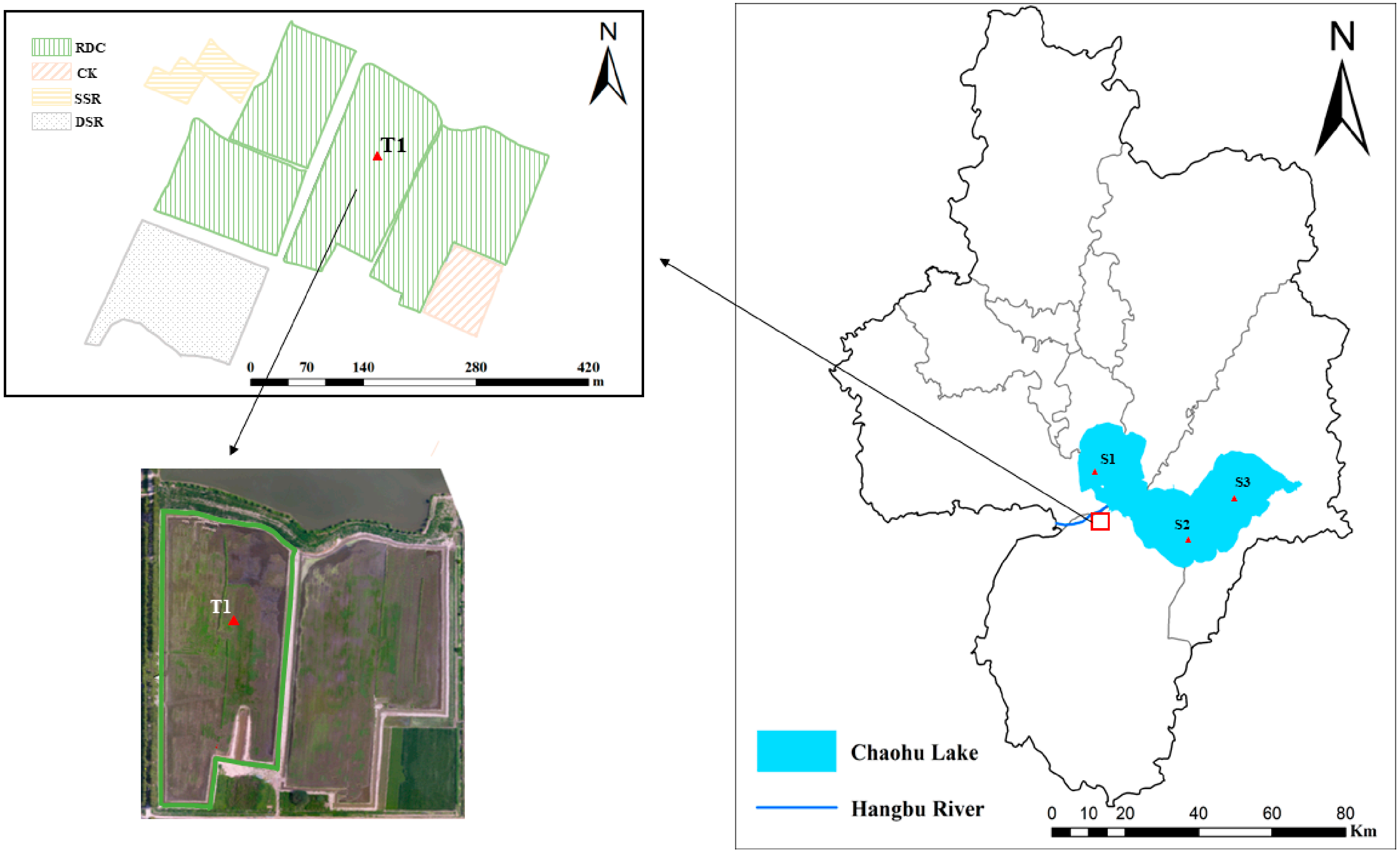
RDC
CK
SSR
DSR
N
T1
0 70 140 280 420
m
T1
N
S1
S2
S3
Chaohu Lake
Hangbu River
0 10 20 40 60 80
Km

Figure S1. The sampling sites in Chaohu Lake (S1-S3) and the paddy soil (T1).

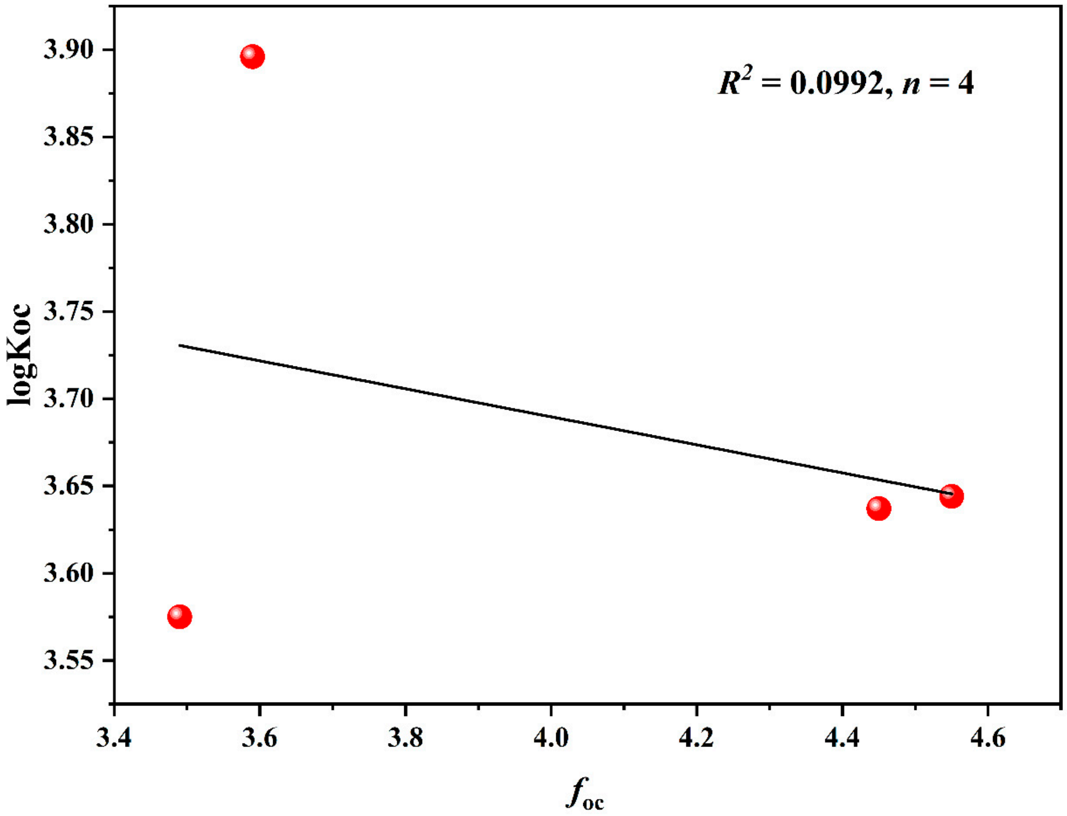

Figure S2. The correlations between f_{oc} and logK_{OC} of different sediments and soil.

Table S1. Occurrence of PCDTs in various environmental matrices.

Country	Environmental matrices	Detected congeners	Range (total)	The dominant congener
America[1]	Sediment	PCDT	——	——
Switzerland[2]	Fly ash	Tetra- and Penta-CDTs	55 $ng\cdot g^{-1}$	2,3,7,8-Tetra-CDT
America[3]	Crabs and lobsters	Tri-, Tetra- and Penta-CDTs	140-8300 $pg\cdot g^{-1}$	2,4,6,8-Tetra-CDT
Japan[4]	Nose soil	Tetra-, Penta-, Hexa-, Hepta- and Octa-CDTs	610-2900 $pg\cdot g^{-1}$ (6550 $pg\cdot g^{-1}$)	——
Japan[4]	Hachiouji soil	Tetra-, Penta-, Hexa- and Hepta-CDTs	64-1700 $pg\cdot g^{-1}$ (4184 $pg\cdot g^{-1}$)	——
Japan[4]	Municipal waste fly ash	Tetra-, Penta- and Hexa-CDTs	440-480 $pg\cdot g^{-1}$ (1380 $pg\cdot g^{-1}$)	——
China[5]	Sediment	14 congeners[5]	0.40-3.55 $ng\cdot g^{-1}$ (16.69 $ng\cdot g^{-1}$)	1,2,3,4,7-Penta-CDT
China[5]	SPM	14 congeners[5]	0.38-2.95 $ng\cdot g^{-1}$ (15.1 $ng\cdot g^{-1}$)	1,3,9-Tri-CDT

Table S1 (continued)

China[5]	Water	14 congeners[5]	0.34-2.61 ng·g^{-1} (13.8 ng·g^{-1})	1,3,9-Tri-CDT

[5] Including DT, 2-Mono-CDT, 4-Mono-CDT, 1,6-Di-CDT, 1,8-Di-CDT, 2,7-Di-CDT, 1,3,9-Tri-CDT, 1,2,6,7-Tetra-CDT, 1,3,6,8-Tetra-CDT, 2,3,7,8-Tetra-CDT, 1,2,3,4-Tetra-CDT, 1,2,3,4,7-Penta-CDT, 1,2,4,6,7,9-Hexa-CDT, and 1,2,3,4,6,7,9-Hepta-CDT.

Table S2. The method detection limits (MDLs) and recoveries of 2,3,7,8-TCDT in sediments and soil.

**Soil / Sediment**	**Method detection limits ($\mu g \cdot g^{-1}$ d.w.)**	**Recoveries (%)**
Paddy soil	0.15	106.9±9.35
Western of Chaohu Lake	0.25	85.2±8.82
Middle of Chaohu Lake	0.45	92.4±6.47
Eastern of Chaohu Lake	0.30	95.5±7.73

References

1. Peterman, P.H.; Smith, L.M.; Stalling, D.L.; Petty, J.D. Identification of chlorinated biphenylenes and other polycyclic aromatic compounds formed from the incineration of PCB-dielectric fluids at a capacitor plant's disposal site. In Proceedings of the 34th Annual Conference on Mass Spectrometry and Allied Topics, Cincinnati, OH, USA, 8 June 1986; pp. 486–487.
2. Buser, H.R.; Dolezal, I.S.; Wolfensberger, M.; Rappe, C. Polychlorodibenzothiophenes, the sulfur analogs of the polychlorodibenzofurans identified in incineration samples. *Environ. Sci. Technol.* **1991**, *25*, 1637–1643.
3. Buser, H.R.; Rappe, C. Determination of polychlorinated dibenzothiophenes, the sulfur analogues of the polychlorinated dibenzofurans, using various gas chromatographic/mass spectrometric techniques. *Anal. Chem.* **1991**, *63*, 1210–1217.
4. Nakai, S.; Kishita, S.; Nomura, Y.; Hosomi, M. Polychlorinated dibenzothiophenes in Japanese environmental samples and their photodegradability and dioxin-like endocrine-disruption potential. *Chemosphere*, **2007**, *67*, 1852–1857.
5. Zhu, Z.Q.; Dai, Y.Y.; Zhang, R.; Shi, J.Q.; Zhang, X.S.; Liu, B.X.; Feng, M.B. Occurrence, distribution and partitioning of polychlorinated dibenzothiophenes (PCDTs) in Chaohu Lake, Southeast China. *Environ. Pollut.* **2021**, *277*, 116751.
